# Safety and Effectiveness of a Novel Fluoroless Transseptal Puncture Technique for Lead-free Catheter Ablation: A Case Series

**DOI:** 10.19102/icrm.2020.110405

**Published:** 2020-04-15

**Authors:** Tariq Salam, Lane Wilson, Sara Bohannan, Michael Morin

**Affiliations:** ^1^Pulse Heart Institute, MultiCare Health System, Tacoma, WA, USA

**Keywords:** Atrial fibrillation, catheter ablation, electroanatomic mapping, fluoroless, transseptal puncture

## Abstract

Increasing awareness of the health risks associated with the exposure of patients and staff in the catheterization laboratory to radiation has encouraged the pursuit of efforts to reduce the use of fluoroscopy during catheter ablation procedures. Although nonfluoroscopic guidance of ablation catheters has been previously described, transseptal access is still perceived as the last remaining barrier to completely fluoroless ablations. This study examined the safety and effectiveness of transseptal puncture and radiofrequency (RF) catheter ablation using a completely fluoroless approach. Three hundred eighty-two consecutive cases that had undergone completely nonfluoroscopic RF catheter ablation were evaluated. Ablation procedures were performed for atrial fibrillation, atrial flutter, atrioventricular reentry tachycardia, and pulmonary vein complex/ventricular tachycardia. Transseptal puncture and RF ablation were conducted under three-dimensional electroanatomic mapping and intracardiac echocardiography image guidance. Fluoroless transseptal puncture and catheter ablation were completed successfully in all cases, with no intraoperative complications. One patient required minimal use of fluoroscopy to visualize sheath advancement through an existing inferior vena cava filter. Procedural time was approximately 2.2 hours from transvenous access until case conclusion; transseptal access was obtained within 28 minutes of procedure initiation. Arrhythmia was found to recur in 27% of cases on average three months after the procedure. We demonstrate the safety and effectiveness of a completely fluoroless transseptal puncture and RF ablation technique that eliminates radiation exposure and enables complex electrophysiology procedures to be performed in a lead-free environment.

## Introduction

Fluoroscopic guidance is routinely used in electrophysiology procedures, such as catheter ablation treatments for cardiac arrhythmias, to facilitate the delivery of catheters and other devices. Although the evolution of X-ray technologies has enabled highly accurate imaging of complex anatomies, such imaging also exposes patients and staff to harmful radiation. Conventional catheter ablation exposes patients to approximately 15 mSv of radiation, equivalent to 750 chest X-rays,^1^ presenting a risk of fatal malignancies^2,3^ or skin injuries that may occur after merely 60 minutes of low-level fluoroscopy.^1^ Although the recent adoption of fluoroscopy with an ultralow frame rate for ablation procedures has reduced patient radiation exposure to as low as 0.45 mSv,^4^ operators and staff still face a significant lifetime cumulative effect of radiation. Occupational radiation exposure of cardiac electrophysiologists, which is estimated to be three times greater than that of diagnostic radiologists and nuclear medicine professionals, averages approximately 5 mSv per year^5^ (i.e., equivalent to 250 chest X-rays). Consequently, electrophysiology staff have an elevated risk of developing brain tumors^6^ and cancers.^5^ A differential radiation dose distribution has been reported whereby head exposure is 10 times greater than whole-body exposure,^7^ with a greater incidence of left-sided malignancies,^8^ including breast cancer^9^ and brain tumors,^10^ consistent with the observed proximity of the operator to the primary X-ray beam and scatter radiation from the patient. Although lead apparel is intended to protect staff from the effects of radiation exposure, up to 44% of interventional cardiologists and electrophysiologists who wear these heavy shields experience spine, hip, knee, and ankle pain,^13–15^ affecting work performance.^14,16^

Increasing research examining the exposure of medical staff and patients to radiation during catheter ablation procedures^1,11,12,17–19^ has prompted efforts to reduce radiation using newer X-ray technologies and the optimization of fluoroscope exposure parameters.^11,20^ However, the use of fluoroscopy with an ultralow frame rate, in particular, has been noted to reduce image quality, providing inadequate visualization for less experienced operators.^4^ Several studies have also explored the use of three-dimensional electroanatomic mapping (EAM) for nonfluoroscopic guidance of ablation catheters as well as two-dimensional intracardiac echocardiography (ICE)^21–26^ or transesophageal echocardiography^27^ for transseptal puncture; however, echocardiographic reverberation has been reported to prevent successful double transseptal punctures, necessitating the switch to a single-puncture approach.^28^ Limited visualization of the transseptal apparatus has been associated with reduced transseptal puncture success, requiring a switch over to fluoroscopic guidance in 30% to 60% of cases.^26,29^ Although EAM-guided transseptal puncture has also been reported,^30,31^ data on the safety and effectiveness of these techniques as well as the ability to visualize the transseptal needle in the superior vena cava (SVC) during dropdown and while positioning the transseptal needle on the interatrial septum are limited. Therefore, in the absence of precise three-dimensional visualization of a transseptal needle, fluoroscopy continues to be used during transseptal puncture and remains the last barrier to completely fluoroless cardiac ablations. This study describes a fluoroless method for transseptal puncture and catheter ablation using three-dimensional EAM, demonstrating safety and effectiveness in the largest series of nonfluoroscopic catheter ablation procedures to date.

## Materials and methods

### Study setup

A retrospective analysis was performed involving 382 patients who underwent zero-fluoroscopy radiofrequency (RF) catheter ablation conducted for atrial fibrillation. All procedures were performed consecutively with no exclusions by one operator (T. S.) between February 2015 and September 2018. Diagnosis and referral for catheter ablation were performed based on the standard protocols in practice. Patients were informed about the risks, benefits, and alternatives of nonfluoroscopic catheter ablation prior to obtaining consent. Procedure time was recorded from initial venous access until final sheath removal after completion of catheter ablation. Acute procedural success, determined by the complete electrical isolation of the pulmonary veins as well as significant intraoperative complications (eg, pericardial effusion, cardiac tamponade) and the recurrence of arrhythmia, were evaluated for each patient. Recurrence, defined by the reappearance of cardiac arrhythmia on an electrocardiogram, was assessed at follow-up. This work was approved by MultiCare Health System’s institutional review board as a quality improvement project, without additional ethics requirements.

### Setup for radiofrequency needle visualization

**Figure 1:**
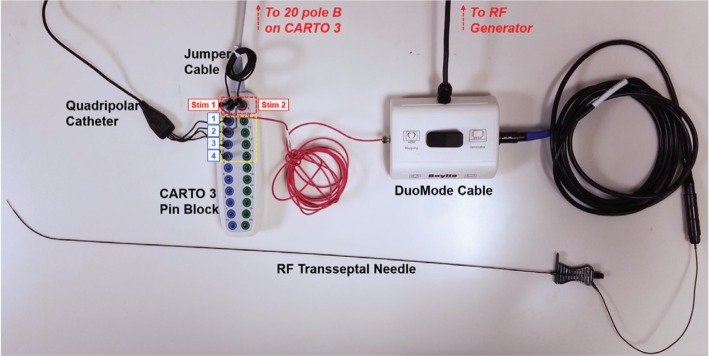
Equipment setup for visualizing the RF transseptal needle. A DuoMode extension cable (Baylis Medical, Montreal, Quebec, Canada) was used to connect the RF needle to the CARTO^®^ 3 pin block.

A dedicated DuoMode extension cable (Baylis Medical, Montreal, Quebec, Canada) was used to connect an RF transseptal needle (NRG Transseptal Needle; Baylis Medical, Montreal, Quebec, Canada) to the CARTO^®^ 3 System (Biosense Webster, Diamond Bar, CA, USA) for visualization in the “mapping” mode. The RF needle, stackable 2-mm pin jumper cable (Biopac Systems, Goleta, CA, USA), and quadripolar catheter (Biosense Webster, Diamond Bar, CA, USA) were connected to the CARTO^®^ pin block, as shown in **Figure 1A**. Preset quadripolar catheter definitions (20B 4F quad 2–5–2 mm fixed) were selected in the CARTO^®^ module version 5 (Biosense Webster, Diamond Bar, CA, USA) to facilitate visualization of the RF needle and esophageal temperature probe using the same pin block. The electrode tip of the RF needle was visualized on CARTO^®^ by showing the extended feature raw electrode data.

### Transseptal puncture and catheter ablation

A short sheath (Avanti Sheath Introducer; Cordis, Hialeah, FL, USA) was inserted to obtain access through the right femoral vein. Under ultrasound guidance (ACUSON X700 Ultrasound System; Siemens Healthineers, Erlangen, Germany), a 0.032-inch guidewire was introduced through the short sheath halfway up the inferior vena cava (IVC) filter to act as a rail for safe advancement of the transseptal introducer and dilator assemblies (Swartz SL0 Transseptal Guiding Introducer; Abbott Laboratories, Chicago, IL, USA). The guidewire and dilator were then removed and the ablation catheter (8-French ThermoCool SmartTouch; Biosense Webster, Diamond Bar, CA, USA) was inserted in the sheath, which could then be used to track up the IVC to the right atrium as indicated by the atrial electrogram recording on the ablation catheter. The ablation catheter was used to construct a matrix of the anatomy from the SVC to the right atrium as well as to mark the location of the SVC, His, and coronary sinus (CS) on the CARTO^®^ map. Special consideration was taken to map high into the SVC to enable later visualization of the needle during dropdown into the right atrium. The CS was mapped to facilitate anatomically guided placement of the CS catheter (7-French DecaNav; Biosense Webster, Diamond Bar, CA, USA). The bundle of His, used as an anatomical reference, was marked by mapping the His signal on the ablation catheter. The SOUNDSTAR ICE catheter (Biosense Webster, Diamond Bar, CA, USA) was used to mark the fossa ovalis on the CARTOSOUND module (Biosense Webster, Diamond Bar, CA, USA) of the mapping system.

**Figure 2:**
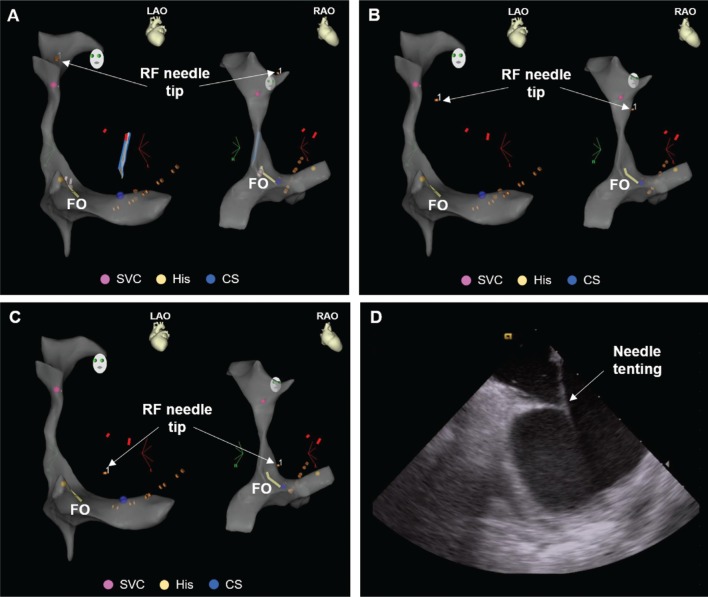
**A–C:** After mapping the right-sided anatomy, CARTO^®^ (Biosense Webster, Diamond Bar, CA, USA) was used to track the descent of the RF needle tip from the SVC to the fossa ovalis. **D:** The location of the fossa ovalis was identified using ICE and superimposed on the CARTO^®^ 3 map. ICE was used in parallel with CARTO^®^ to verify positioning of the needle tip at the fossa ovalis and observe tenting of the interatrial septum prior to RF puncture.

The ablation catheter was used to guide the sheath back to the SVC; the sheath location in the SVC was subsequently confirmed as the proximal poles of the ablation catheter turned black on CARTO^®^ when covered by the sheath. The ablation catheter was replaced by a dilator preloaded with a 0.032-inch guidewire advanced into the SVC. The guidewire was then removed and an RF needle was positioned at a predetermined distance, one thumb-width between the sheath and needle hubs, without exposing the needle tip. The sheath and dilator were then pulled back over the needle to expose the electrode tip just past the end of the dilator to enable signal detection on CARTO^®^ and, therefore, conducted positional tracking of the needle tip as it was dropped down from the SVC toward the right atrium and engaged with the fossa ovalis **(Figure 2)**. ICE was used to verify the needle tip’s position on the fossa ovalis. The interatrial septum was crossed by switching the DuoMode extension cable to the “generator” mode and applying RF energy (one second, pulse mode) through a dedicated RF generator (BMC RF Puncture Generator; Baylis Medical, Montreal, Quebec, Canada). Sheath crossing and positioning as well as the formation of microbubbles in the left atrium were confirmed on ICE before removing the transseptal needle and dilator. A second transseptal puncture was performed using the technique described above. Once the two transseptal access sites were established, the two transseptal sheaths were used to introduce the ablation catheter as well as a mapping catheter (7-French PentaRay NAV Catheter; Biosense Webster, Diamond Bar, CA, USA).

**Figure 3:**
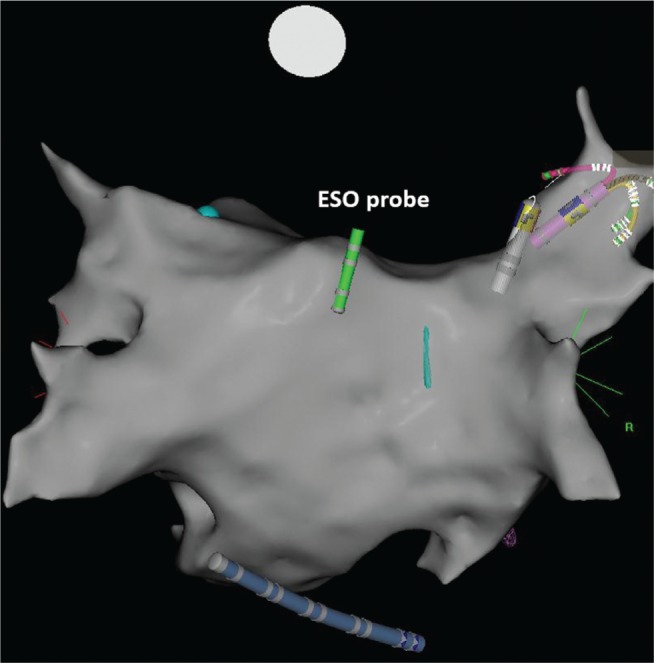
The esophageal temperature probe was attached to a quadripolar catheter to allow visualization on the CARTO^®^ map for real-time positional confirmation during the ablation procedure.

Patients were given uninterrupted oral anticoagulation 24 hours before the procedure and heparin intraoperatively before transseptal access was obtained to maintain an activated clotting time of more than 320 seconds, then subsequently underwent mapping of the left atrium and catheter ablation for pulmonary vein isolation. The esophageal temperature was monitored adjacent to the posterior wall of the left atrium during the ablation procedure using probe (Level 1 Esophageal Stethoscope Temperature Sensor; Smiths Medical, Dublin, OH, USA) attached to the quadripolar catheter using a silk suture to facilitate positional confirmation on CARTO^®^ during the procedure **(Figure 3)**.

## Results

### Patient characteristics

A total of 382 RF catheter ablation procedures were performed on 324 patients with atrial fibrillation (57% paroxysmal, 43% persistent) between February 2015 and October 2018. The mean patient age in the series was 60.7 years ± 13.2 years and 69.4% of the total study population was male. The majority of patients were overweight or obese, with a mean body mass index of 31 kg/m^2^ ± 6.2 kg/m^2^. Baseline patient characteristics are listed in **Table 1**. There was a history of previous transseptal puncture in 30.9% of patients; further, 15.2% of cases within this series were repeat ablation procedures.

### Transseptal puncture

Transseptal access was achieved successfully in all cases without any significant intraoperative complications such as pericardial effusion or cardiac tamponade **(Table 2)**. Double transseptal puncture was performed in 94% of cases. Fluoroscopy was not used to guide transseptal access in any of the cases. Only one patient with an IVC filter required 2.2 minutes of fluoroscopy to visualize sheath advancement toward the SVC. Transseptal access, including right atrial mapping, was achieved within 28 minutes ± 15 minutes of initial femoral vein access. Where double transseptal puncture was required, a second instance of transseptal access was obtained within less than five minutes.

### Ablation

Acute procedural success was achieved in 100% of cases without the use of fluoroscopy during the ablation step **(Table 3)**. RF ablation for atrial fibrillation was performed in 325 cases (ie, 85% of all cases). The atrial flutter line was ablated as the primary or secondary arrhythmia in 51 cases (13%). Ablation for primary or secondary atrioventricular reentrant tachycardia was performed in 25 cases (6.5%). A total of eight ablations (2.1%) were performed to target premature ventricular complexes or ventricular tachycardia. The average total procedure time for all arrhythmias, from transvenous access to final sheath removal, was 134.7 minutes ± 33.5 minutes. All procedures were concluded without any significant intraoperative complications.

The majority of patients (90%) were seen during follow-up at a mean of three months ± one month after the procedure. A recurrence of arrhythmia was noted in 26.7% of cases.

## Discussion

To our knowledge, this is the largest study yet demonstrating the safety and effectiveness of a completely nonfluoroscopic guidance technique for both transseptal puncture and catheter ablation for atrial fibrillation. Our center had previously implemented a standardized protocol for left heart access using a conventional mechanical needle approach. In an effort to eliminate radiation exposure and establish a lead-free cardiac catheterization laboratory environment and, given an ongoing internal quality improvement initiative, we herein evaluated a new transseptal technique using the combination of an RF transseptal needle, three-dimensional EAM, and ICE. In accordance with research by Winkle et al.,^32^ we found that the incremental cost of an RF transseptal needle can be offset by its ease of use and the ability to perform transseptal puncture safely and with precision without fluoroscopy.

By visualizing the rounded electrode tip of an RF needle on EAM, we were able to track the positioning of the needle as it was dropped down from the SVC to the fossa ovalis. Although conventional mechanical needles may also be visualized on EAM systems by extending the needle tip beyond the end of the dilator, it is not possible to safely maintain the sharp needle tip exposed to facilitate visualization while dropping down from the SVC and locating the fossa ovalis. Furthermore, since the entire shaft of a mechanical needle is not electrically insulated, the positioning of the tip, as interpreted by the EAM system, may not be as precise. ICE imaging can also enable visualization of a mechanical needle without fluoroscopy; however, it has been reported to compromise the ability to perform double transseptal punctures due to echocardiographic reverberation, thereby necessitating catheterization through a single puncture site,^28^ which, in turn, has been linked to increased risks of silent cerebral events^33^ and persistent atrial septal defects^34^ relative to double transseptal access. The use of a dedicated RF transseptal needle not only enables a fluoroless technique but also improves transseptal puncture success in a wider range of patient septal anatomies that may otherwise necessitate makeshift and not fully characterized use of electrocautery with a mechanical needle and prompt associated risks of injury congruent with an electrified sharp needle tip.

Using the technique described in this study, we experienced 100% success in performing a double transseptal puncture without any fluoroscopy regardless of septal anatomy or history of prior transseptal catheterization. In comparison, a recent study describing a method for visualizing a sharp mechanical transseptal needle on EAM reported unsuccessful transseptal puncture in 10% of patients as well as one case of cardiac tamponade.^30^ Further, the use of heavy protective lead apparel was not necessary with our technique, providing relief to the operator and electrophysiology staff. The only application of fluoroscopy in this case series was to confirm the location of an IVC filter in one patient during sheath advancement; however, subsequent transseptal puncture and catheter ablation were performed fluorolessly. The first transseptal puncture, including mapping of the right-sided cardiac anatomy, was typically achieved within 28 minutes of initial venous access. Then, the second transseptal puncture subsequently took place within five minutes of the first puncture. These times are comparable to those seen with fluoroscopy-guided RF transseptal puncture, which has been reported to require up to 27 minutes from femoral vein access, and are improved relative to the use of conventional mechanical needles with fluoroscopy.^35^ All procedures were completed successfully with no significant intraoperative complications within an average of 2.25 hours, similar to as previously reported.^21–23,27^ Postoperative evaluation at an average of three months after ablation indicated a 27% rate of cardiac arrhythmia recurrence, in line with other studies.^36,37^ In our center’s experience, the technique described in this study improved procedural efficiency by approximately 15 minutes, which, combined with recent efforts to shift final sheath removal to the postanesthesia care unit, can lead to saving up to 30 minutes per procedure, allowing for more efficient procedure room turnaround between cases and reducing the necessity for staff to work overtime. Furthermore, our findings suggest that this fluoroless catheter ablation technique can be combined with operational efficiency initiatives without encumbering overall quality improvements.

Of note, this is a single-center retrospective analysis that is subject to inherent limitations. This study was not intended to be a comparative assessment against conventional fluoroscopic methods, which would, typically, require a randomized controlled trial. Furthermore, a single operator performed all procedures after full adoption of the fluoroless technique using the CARTO^®^ 3 System (Biosense Webster, Diamond Bar, CA, USA). Additional studies are needed to compare the safety and effectiveness of conducting catheter ablation using fluoroless versus conventional fluoroscopy approaches with different levels of operator experience as well as to demonstrate the applicability and utility of this technique in conjunction with other EAM systems. Although early arrythmias at three months postablation have been correlated with long-term recurrence at one year, extended arrhythmia monitoring beyond the initial three-month follow-up period is needed to confirm the efficacy of catheter ablation.^38^ The use of ICE may be perceived as an added cost in centers that do not routinely use ICE; however, the use of EAM alone to visualize the transseptal needle has been associated with unsuccessful transseptal puncture and cardiac tamponade.^30^ Detailed economic analysis is also needed to quantify time and cost savings associated with procedural success using the described fluoroless technique and workflow modifications.

## Conclusion

This study describes a technique for zero-fluoroscopy transseptal puncture and catheter ablation for atrial fibrillation. We demonstrated the safety and efficacy of this approach in a large case series without affecting procedure time or outcomes. This completely fluoroless technique eliminates the health risks associated with exposure of both patients and staff to radiation, allowing complex electrophysiology ablations to be performed in a lead-free environment while potentially improving operational efficiency and reducing the physical burden on operators and staff.

## Figures and Tables

**Table 1:** Baseline Patient Characteristics (n = 324)

Average age (years)	60.7 ± 13.2
Male sex (%)	69.4
Average body mass index (kg/m^2^)	31.0 ± 6.2
AF classification (%)	
Paroxysmal	57
Persistent	43
Hypertension (%)	47.5
Diabetes (%)	13.6
Coronary artery disease (%)	15.1
Average ejection fraction (%)	56.0 ± 7.9
Oral anticoagulant use (%)	90.2
History of prior transseptal puncture (%)	30.9

**Table 2:** Transseptal puncture profile

Parameter	Number
Total number of punctures	742
Puncture success (%)	100
Complications (%)	0
Fluoroscopy time during puncture (min)	0 ± 0
Time to transseptal access (min)	27.8 ± 15.1

**Table 3:** Overall Procedural Information

Parameter	Number
Arrhythmia type	
Atrial fibrillation	325
Atrial flutter line	51
AVRT	25
PVC/VT	8
Other	4
Procedure time (min)	
Overall	134.7 ± 34.5
Fluoroscopy	0 ± 0.1
Atrial fibrillation	134.0 ± 30.5
Atrial flutter line*	152.3 ± 43.7
AVRT*	120.7 ± 49.0
PVC/VT	161.0 ± 31.2
Other	153.6 ± 22.5
Acute ablation success (%)	100
Follow-up time (months)	3.0 ± 1.0
Recurrence (% yes)	26.7

AVRT: atrioventricular reentrant tachycardia; PVC/VT: premature ventricular contraction/ventricular tachycardia.

*Includes arrhythmias treated primarily and secondarily.
